# Using audience segmentation to identify implementation strategies to improve PrEP uptake among at-risk cisgender women: a mixed-methods study protocol

**DOI:** 10.1186/s43058-023-00518-z

**Published:** 2023-11-17

**Authors:** Jennifer A. Pellowski, Devon M. Price, Arielle Desir, Sarit Golub, Don Operario, Jonathan Purtle

**Affiliations:** 1 Department of Behavioral and Social Sciences, Brown University School of Public Health, International Health Institute, 121 South Main Street, Providence, RI 02903 USA; 2 Department of Psychology, Hunter College & Graduate Center of the City University of New York, 695 Park Avenue, New York, NY 10065 USA; 3 Department of Behavioral and Social Sciences, Brown University School of Public Health, 121 South Main Street, Providence, RI 02903 USA; 4 Department of Behavioral, Social, and Health Education Sciences, Emory University Rollins School of Public Health, 1518 Clifton Road, Atlanta, GA 30322 USA; 5 Department of Public Health Policy & Management, Global Center for Implementation Science, New York University School of Global Public Health, 708 Broadway, New York, NY 10003 USA

**Keywords:** PrEP, Cis women, Implementation strategies, Audience segmentation

## Abstract

**Background:**

In the USA, 19% of new HIV infections occur among cisgender women (cis women); however, only 10% of eligible cis women have been prescribed pre-exposure prophylaxis (PrEP) for the prevention of HIV infection (an evidence-based intervention). A fundamental challenge for expanding HIV prevention to cis women is ensuring implementation strategies are tailored to the various healthcare settings in which cis women seek care and the heterogeneous providers nested within these settings. This project’s specific aims are to (1) explore clinician-level characteristics and organizational climate factors that are related to variability in adoption of PrEP service delivery as an evidence-based intervention for cis women; (2) identify latent audience segments of women’s health providers as the related to PrEP acceptability, adoption, and maintenance and analyze demographic correlates of these segments; and (3) identify audience segment-specific implementation strategies to facilitate the adoption of PrEP as an evidence-based intervention among at-risk cis women.

**Methods:**

Using the i-PARIHS framework, this mixed-methods study examines three domains for guiding audience segmentation to facilitate PrEP implementation for cis women: innovation (degree of fit with existing practices, usability), recipient beliefs and knowledge and context factors (organizational culture, readiness for change), needs to determine appropriate facilitation methods. To achieve aim 1, qualitative interviews will be conducted with PrEP-eligible cis women, women’s health providers, and other key stakeholders. Aim 2 will consist of a quantitative survey among 340 women’s health providers. Latent class analysis will be used to facilitate audience segmentation.

To achieve aim 3, a panel of 5–8 providers for each audience segment will meet and engage in iterative discussions guided by Fernandez’s implementation mapping to identify (1) implementation outcomes and performance objectives, determinants, and change objectives and (2) determine and refine of implementation strategies for each audience segment.

**Discussion:**

This exploratory mixed methods study will provide an empirical foundation to inform the development implementations strategies aimed at increasing PrEP delivery to cis women among heterogenous groups of providers.

Contributions to the literature

- Clinical providers are the target of many implementation strategies; however, these strategies are rarely tailored for the heterogenous characteristics of different types of providers and their clinical settings that may impact acceptability, adoption, and sustainment.
- Audience segmentation has been used to tailor dissemination (i.e., communication) strategies, more so than implementation strategies.
- This protocol demonstrates how audience segmentation can be used to characterize different groups of potential adopters and facilitate development of tailored implementation strategies.

## Background

In the USA, 19% of new HIV infections occur among cisgender (cis) women; however, only 10% of cis women who could benefit from pre-exposure prophylaxis (PrEP) for the prevention of HIV infection received a prescription [1]. There remains a large health disparity in access to this medication; cis men who have sex with men (MSM) who would benefit from PrEP are 4 times as likely to be on medication compared to cis women who would benefit from the medication [2]. Unlike MSM or trans women, cis women are likely to seek sexual health services from practices that focus on reproductive health and family planning, such as OB/GYN healthcare practices and family medicine/primary care [3–6]. However, women’s healthcare providers are heterogenous in terms of training, clinical environment, knowledge, and attitudes. There are limited data about how implementation strategies may be tailored to account for this heterogeneity among providers with respect to PrEP prescription [5, 7–9]. Furthermore, current methods in implementation science have not thoroughly specified approaches for identifying heterogeneity among potential intervention adopters across settings in order to determine how implementation strategies could be most effectively tailored for different groups. Recommendations for tailoring implementation strategies exist but are not focused on tailoring based on heterogeneity in adopter characteristics [10]. Recommendations for tailoring dissemination (i.e., communication) strategies to account for audience heterogeneity exists but do not consider a wider range of implementation strategies beyond those focused on dissemination [11–15].

To advance research on audience segmentation and implementation strategies, we will use an exploratory, sequential mixed methods design (QUAL → QUANT) that will apply audience segmentation methods to inform how implementation strategies may for tailored to increase the reach of PrEP among cis women. This project uses the i-PARIHS framework [16] to inform data collection about the heterogeneous perceptions with regard to: the PrEP *innovation* (degree of fit with existing practices, usability), *recipient* beliefs and knowledge, and *contextual* factors (organizational culture, readiness for change) to determine appropriate *facilitation methods*. More broadly, this project will contribute to empirical research about implementation strategies tailoring through the novel integration of audience segmentation methods.

### Study aims

This study has three aims:

- *Aim 1: Explore clinician-level characteristics and organizational climate factors that are related to variability in PrEP service delivery for cis women*. We will conduct in-depth interviews with sexual health providers, cis women eligible for PrEP, and other key stakeholders (e.g., women’s sexual health experts, public health officials, PrEP navigators, specialists, clinical case workers) to identify factors that may be associated with the latent audience segments that will be quantitatively derived in aim 2.
- *Aim 2: Identify latent audience segments of women’s health providers and analyze demographic correlates of these segments to inform the development of PrEP implementation strategies for providers within segments*. We will conduct quantitative surveys in a sample of sexual health providers (*N* = 340) with prescribing authority recruited from clinics in the Northeast and Southern US, in order to identify patterns in PrEP-relevant attitudes (e.g., HIV stigma, PrEP perceived as within scope of practice), PrEP knowledge, and organizational climate (e.g., readiness for change). Utilizing these data, we will use latent class analysis to identify audience segments.
- *Aim 3: Identify audience segment-specific implementation strategies to facilitate the implementation of PrEP among at-risk cis women*. Based on information gathered in aims 1 and 2, we will construct a menu of strategies for engaging with providers nested within clinics for each audience segment. Using expert panels of providers representing each audience segment, this menu will be refined into targeted implementation strategies to be tested in a subsequent study.

### Provider focused interventions for PrEP uptake

A major gap in the efforts to expand access to PrEP among cis women is the lack of implementation of PrEP programs in settings where women traditionally seek sexual healthcare [4–6, 8]. Cis women who had a recent birth control visit or sought abortion care are at greater likelihood for being indicated for a PrEP prescription, further indicating women’s healthcare practices as being an important place of intervention to expand access to cis women who would benefit from PrEP [17]. However, cis women are often not offered PrEP and are less likely to be tested for HIV in these settings [18]. As OBGYN practices become more apparent as a means of expanding access of PrEP to cis women [19, 20], implementation science is needed to prepare for this expansion in efficient and efficacious ways.

Implementation science has been critical to the rollout of PrEP among MSM and trans women in rapidly expanding access to the medication across the USA [21, 22]. One of the earliest and most successful PrEP implementation efforts included clinics providing patients with the medication for free along with a one-on-one counseling session by a knowledgeable and culturally competent healthcare provider [23]. In this study, the overwhelming majority of participants continued PrEP throughout all 12 months of observational follow-up and noted that key facilitators of their retention in care included the minimal financial burdens (of both the medication and clinical care) as well as the one-on-one counseling by a healthcare provider [23]. Efforts to expand access to PrEP in the South have included identifying cultural stigma around seeking and utilizing the medication by both patients and their providers, and empowering clinics to use creative techniques in supporting patients’ navigation of their more conservative sexual health policy landscape and patients were more likely to start and stay on PrEP if their providers were encouraging of their use [24].

More recently, PrEP implementation efforts for gay and bisexual men, as well as transgender women, have focused on reducing racial and ethnic disparities of those who utilize the medication [25]. These strategies include redefining clinical PrEP eligibility assessment to expand access to those previously not considered “at-risk,” de-emphasizing risk perception as a strategy to increase demand, altering current clinical guidelines to make PrEP follow-up less onerous, and focusing directly on strategies to reduce the cost of PrEP medication [25]. Taken together, the key lessons from past PrEP implementation efforts among gay and bisexual men, and transgender women, have focused on structural changes at the clinics where these populations receive their care as well as intervening on the providers themselves to facilitate a pleasurable and healthy sex life for their patients.

There have been some PrEP implementation efforts geared towards the unique needs of cis women, but much of this work has taken place in the context of countries in Africa, with some limited work in the USA [26–28]. In Kenya and South Africa, implementation strategies utilized for increased uptake of PrEP among cis women have included not only making women aware of their potential risk for HIV but simultaneously training their sexual healthcare providers to be aware of that risk as well [26, 27]. In the USA, similar themes have been identified; women living in Mississippi similarly reported low perceptions of being at-risk for HIV [28]. Their providers similarly seemed to be concerned with preventing pregnancy and STIs like gonorrhea and chlamydia but were not as concerned about the chance of their patients being exposed to HIV [28]. When a provider was aware of the heightened risk of HIV among Black women in the South, they struggled to provide their patients with a PrEP prescription due to financial and time burdens of the medication, including repeated blood work every 3 months [28]. The providers identified that the lives of Black women in Mississippi were often very busy, and their own health needs tended to be deprioritized compared to their care-giving responsibilities and work [28]. These social realities highlight the gap between PrEP clinical protocols and implementation into the lives of women who would most benefit from utilization of PrEP.

### Audience segmentation for PrEP uptake

Utilized heavily in marketing research, audience segmentation is a process where a population is divided into subgroups based on common characteristics such as product usage, communication style, or demographics, in order to tailor messages for greater impact [29]. Messages that are tailored to audience segments are typically more effective than non-tailored messages [30, 31]. Empirically, this approach has also been used to inform *dissemination* strategies in women’s health research including preconception health [32], mammogram adoption [33], and tanning bed use [34]. Dudley et al. [35] utilized audience segmentation to determine typologies of pregnant women around vaccination attitudes (vaccine supporters, vaccine acceptors, vaccine skeptics) to facilitate the tailoring of educational interventions to increase vaccination for these differing subgroups. This approach has also been used with to identify social marketing segments among adolescent girls in South Africa based on lifestyles, values, and sexual practices, which has application to HIV prevention marketing [36].

Although the use of marketing techniques has been a cornerstone for HIV prevention messaging since the start of the epidemic [37], only a few studies have used audience segmentation PrEP uptake. Utilizing demographic segmentation, Marshall et al. [38] describe targeted campaigns used by departments of health social marketing campaigns to racial/ethnic and sexual/gender minorities but do not identify these segments through empirical analysis. Conversely, Bass et al. [39] use empirical clustering segmentation to identify optimal dissemination messages for PrEP uptake among transwomen; utilizing k-means cluster analysis, researchers identified three unique clusters marked by varying levels of trust in the transgender community, trust in information about PrEP, and receipt of PrEP education [39]. To our knowledge, audience segmentation has not yet been used for PrEP uptake among cis women, who have distinct needs from trans women and MSM. Importantly, marketing techniques for PrEP have been targeted towards the users and not at providers. Providers’ awareness of and willingness to prescribe PrEP to eligible patients is essential to increasing the uptake of PrEP [40–42]. This represents a missed opportunity to harness the effectiveness of marketing techniques, such as audience segmentation, to improve provider awareness and comfort prescribing PrEP outside of LGBT health centers.

### Audience segmentation as a novel approach for identifying implementation strategies

While audience segmentation is widely recognized as having potential to inform the development of dissemination strategies (which typically involve asynchronous communication of evidence and are focused on outcomes such as knowledge, attitudes and intentions), the practice has not been widely applied to inform the development of implementation strategies (which involve a much wider range of strategies and are focused on outcomes related to behavior change [11–15, 43, 44]. Furthering understanding of how audience segmentation can be applied to inform the development of implementation strategies would complement prior work focused on tailoring implementation strategies for different contexts and provider types [10]. While audience segmentation has been used in implementation research to identify segments of providers, policymakers, and other practices audience, none of this prior work has focused on PrEP [45–47].

There is likely substantial variability in the types of implementation strategies needed to account for heterogeneity among cis women’s health providers in order to increase prescription of PrEP for eligible cis women. Such heterogeneity likely spans domains such as the clinic settings in which they work [5, 7–9]. Furthermore, implementation strategies for providers may vary depending on individual attitudes towards PrEP as an HIV prevention strategy for cis women, perceived scope of practice, and knowledge about cis women’s specific considerations for PrEP. Thus, audience segmentation provides a vehicle for understanding this variability in order to categorize cis women’s health providers based on their characteristics and organizational climates and match these sub-populations to appropriate implementation strategies to increase the prescription of PrEP among cis women.

## Methods

**Fig. 1:**
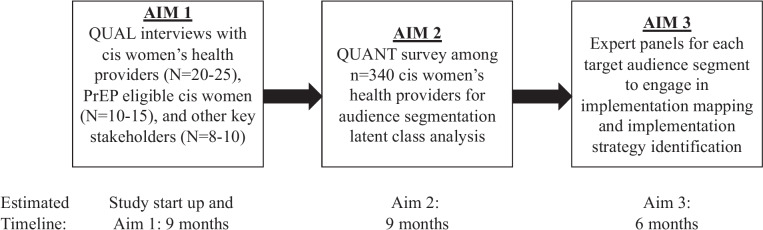
Study process

This exploratory project uses a sequential mixed method (QUAL → QUANT) design with three distinct phases (Fig. 1). This project focuses on PrEP uptake among cis women’s health providers, broadly defined as individuals currently working in clinical capacity with cis women and have prescriptive authority (i.e., gynecologist/obstetrician, primary care providers, nurse practitioners). The research team consists of experts in women health, HIV prevention, PrEP, and implementation science.

### Conceptual frameworks

This project utilizes two implementation science frameworks to guide the study of potential segments of cis women’s health providers: (1) the revised Promoting Action on Research Implementation in Health Services (i-PARIHS) framework [16] and (2) Fernandez et al.’s Framework for Implementation Mapping [48]. The i-PARIHS Framework specifies a two-stage process in which *innovation* (degree of fit with existing practices, usability), *recipients* (provider beliefs and knowledge), and *context* (organizational culture) are measured, evaluated, and aggregated and then used to determine appropriate *facilitation methods* (in this case, PrEP implementation guidance for each clinical audience segment). For the current project, aims 1 and 2 will focus on innovation, recipients, and context, and aim 3 focuses on facilitation method determination (i.e., implementation strategies). In addition to the i-PARIHS framework, this project also utilizes Fernandez et al.’s Framework for Implementation Mapping, which outlines a systematic, iterative process of developing implementation strategies to improve implementation outcomes and is comprised of 5 “tasks” [48]; the current project focuses on the first 3 tasks of this framework. Aims 1 and 2 provide an assessment of characteristics related to PrEP among cis women’s health provider audience segments (Task 1: Conduct a needs and assets assessment and identify adopters and implementers). Aim 3 will produce PrEP implementation protocols tailored to each provider audience segment to be tested in a subsequent evaluation (Tasks 2: Identify adoption and implementation outcomes, performance objectives, and determinants; create matrices of change and Task 3: Choose theoretical methods, select or create implementation strategies).

### Aim 1 methods

Aim 1 methods consist of in-depth qualitative interview with the target patient population (i.e., PrEP-eligible cis women), the target implementation population (i.e., cis women’s health providers in the USA), and other key stakeholders (e.g., clinical administrators, non-governmental organization representatives, PrEP navigators,) to characterize experiences, attitudes, and interactions related to the delivery of PrEP services for at-risk cis women and information about organizational climates.

#### Interview samples, recruitment, and data collection

Cis women’s health providers will be recruited online and through partner healthcare centers [49]. Eligibility criteria include (a) 18 years or older and (b) currently working in clinical capacity with cis women and have prescriptive authority (i.e., gynecologist/obstetrician, primary care providers, family medicine, nurse practitioners). We will use purposive sampling [50, 51] to recruit providers across key dimensions related to PrEP prescribing including, but not limited to, prior knowledge of PrEP for cis women (not very/somewhat knowledgeable vs. very knowledgeable about PrEP), previous prescribing practices of PrEP (never vs. have prescribed prior), and organizational environment (women’s health focused office/center vs. LGBT-focused office/center).

PrEP-eligible cis women will be recruited online and through partner organizations. Eligibility criteria for cis women are as follows: (a) 18 years or older, (b) self-reported cis woman, (c) eligible for PrEP based using current U.S. Centers for Disease Control and Prevention guidelines [52], and (d) speaks English or Spanish. Our goal is to recruit both women who current or have previously used PrEP and those who PrEP naïve.

Other key stakeholders will be recruited from our partner organizations and those who consulted as stakeholders in the previous grants of the Co-Is. Eligibility criteria for health officials are as follows: (a) 18 years or older and (b) 2 + years of experience working in healthcare, healthcare administration, or NGO administration. We intend to sample from a wide variety of organizations and roles including administrators/directors of health centers, PrEP navigators, and NGO/LGBT health center administrators in the USA. All participants will complete informed consent prior to enrollment. All interviews will be conducted online via video-conferencing software, and voice-only recordings will be retained.

Interview content will be informed by the i-PARIHS framework, and interviews will examine provider knowledge and attitudes as well as the unique organizational factors that providers, health officials, and women eligible for PrEP experience. Cis women’s health providers will be asked about:

- Knowledge and awareness of PrEP for cis women and whether they have *adopted* PrEP into their practice already
- Perceived *acceptability* of PrEP among their patients
- *Appropriateness* of PrEP for ciswomen and how they view PrEP within the larger landscape of HIV prevention, prevention of other STDs, and contraceptives
- Perceived *feasibility* and/or concerns related to implementing PrEP for cis women; how innovations in PrEP technologies (e.g., injectable cabotegravir [53]) may impact feasibility and acceptability among patients
- Perceptions of social norms around the prescription of PrEP for cis women by other similar providers (perceived *penetration*)
- Knowledge and concerns about *cost*/payment for PrEP
- Perceptions of current organizational climate including support for PrEP, readiness for change, and perceived clinic-level barriers that could impact *sustainability*

Cis women on PrEP will be asked about their knowledge of PrEP, concerns related to PrEP including provider concerns, any personal experiences with obtaining PrEP including provider and clinic level observations, and recommendations for provider and systems-level improvements. Other key stakeholders will be asked about the larger HIV prevention and PrEP landscape in the USA, current access to PrEP for cis women, structural factors associated with access to PrEP, PrEP uptake strategies that have been used for cis gender gay men and trans gender women, and what strategies have failed to engage cis women.

#### Data processing and analysis

Audio recordings will be transcripted using a secure, professional transcription service. Transcripts will be anonymized during transcription, and all transcripts will be reviewed by the study team to ensure anonymization prior to analysis.

The focus of data analysis is to identify themes related to heterogeneous provider attitudes, knowledge, experiences, and organizational climates that may be indicative of audience segments to inform the development of a survey to empirically determine the audience segments in aim 2. To accomplish this, we will use an inductive thematic analysis [54, 55] in conjunction with “sensitizing concepts” [56] to guide our analyses. Sensitizing concepts allow researchers to start with a general reference point (i-PARIHS framework) to guide interpretation of empirical data/themes while maintaining the use of inductive analysis to allow themes to emerge from the data [56]. In-depth qualitative data analysis techniques will be used including open coding, axial coding, marginal remarks, comparisons, and memo-writing. The full research team will review the initial results and interrogate the findings, which will result in additional queries and validity checks of the qualitative findings.

The key output of this aim is the identification of clinician-level characteristics (attitudes, beliefs, behaviors, and experiences) and organizational climate factors that may be indicative of audience segments structured by the key constructs proposed by the i-PARIHS framework. Data from the qualitative phase will be used to refine the measures used in the aim 2 provider survey.

### Aim 2 methods

Aim 2 methods consist of a cross-sectional quantitative survey with cis women’s health providers (*N* = 340). The purpose of this phase is to empirically determine latent sub-populations (audience segments) of cis women’s health providers based on provider characteristics and the organizational climates in which they are situated. PrEP-relevant provider characteristics that differentiate audience segments may include PrEP knowledge, HIV-related stigma, comfort in prescribing, perceptions of PrEP in scope of practice, perceived organizational support of PrEP, and/or perceived organizational readiness for change; however, selection of these measures will be finalized following aim 1. Provider demographics and readily available clinic level characteristics will be evaluated as predictors of latent segment membership.

#### Survey sample, recruitment, and data collection

Eligibility criteria for women’s health providers will be (a) 18 years or older, (b) currently working in clinical capacity with cis women and has prescriptive authority (i.e., gynecologist/obstetrician, primary care providers, family medicine, nurse practitioners), and (c) living and working in the USA. Providers will be recruited online (e.g., social media and national organization listservs) and through from our partner healthcare centers through listservs for clinic staff. We will use a two-step recruitment process: first, we will use snowball sampling for general clinical provider recruitment; second, mid-way through the recruitment process, we will investigate the diversity of providers who have completed the survey (particularly with regard to PrEP prescription experience and organization climate [e.g., OB/GYN practices, LGBT clinics, primary care]) and will subsequently use heterogeneity sampling to recruit under-represented provider types to ensure a diverse sample and reduce recruitment bias [57, 58].

All surveys will be conducted online. Potential participants will be directed to an information page about the study prior to screening and consent. The survey will be marketed as a survey on “sexual and reproductive health services and provider needs” and will not explicitly mention PrEP in the marketing to limit sampling bias. Eligibility will be assessed through an online screening tool. If eligible, participants will be asked if they understand the risks and benefits of the study and if they would like to continue. Once they select continue, they will be considered enrolled in the study. Completion of survey instruments will take approximately 15–20 min to complete.

Data collected in these surveys will be used to determine empirical clusters of audience segments as well as predictors of segment membership. Based on previous literature and the team’s experience, we propose the several key constructs (see Table 1, guided by key dimensions from the i-PARIHS framework) [23, 59–62]; however, the survey will largely be informed by the themes that arise from the interviews that take place in aim 1. For example, PrEP-relevant provider characteristics that differentiate audience segments may include PrEP knowledge, HIV-related stigma, comfort in prescribing, perceptions of PrEP in scope of practice, perceived organizational support of PrEP, and/or perceived organizational readiness for change (see Table 1 [23, 59–62]). Data will also be collected on implementation outcomes from provider perspectives as determined to be relevant in aim 1 and may include acceptability, appropriateness, feasibility, adoption, cost, and/or sustainability. Providers will be asked about demographic information including position type, gender, years in practice, and region of country to be used as predictors of audience segments.

**Table 1:** Potential survey measures for aim 2

i-PARIHS construct	Potential items and scales
**Innovation** (e.g., degree of fit with existing practice and values; usability; relative advantage)	Scope of practice: Do you consider PrEP prescription and monitoring within you scope of practice? (Yes/No)
	Comfort: (1) How comfortable do you currently feel prescribing PrEP to MSM? (2) How comfortable do you currently feel prescribing PrEP to cis women? (5-point Likert scale, Very Uncomfortable to Very Comfortable)
	Patient risk: How at risk do you think your general patient population is for HIV? (5-point Likert scale, Not at risk at all to Very High Risk);
	Usability: perceptions of barriers to provision of PrEP, 7 items [23]
**Recipients** (e.g., values and beliefs, skills and knowledge)	PrEP Knowledge, 6 items [59]
	HIV-related stigma among providers, 6 items [60]
	Beliefs, attitudes, and concerns about PrEP, 8 items [61]
	Openness: How open are you to receiving more information and support about the provision of PrEP for cis women? (5-point Likert scale, Not Open at All to Very Open)
	Behavior: Have you ever prescribed PrEP to a cis-gender woman before? (Yes/No)
**Context** (e.g., organizational culture; leadership and management support)	Practice type (family planning, reproductive health, LGBT health)
	PARIHS Contextual Readiness to Change Scale [62]
	Availability of benefits navigation support in organization (Yes/No/Unsure)
	Perceived provider autonomy in clinic [62]
	Perceived clinic team effectiveness [62]

#### Sample size calculation and data analyses

Latent class analysis (LCA) is a method for determining audience segmentation [47, 63] because it allows for individuals to be classified into groups based on their pattern of responses, creating groups of individuals that have similar attitudes, behaviors, and needs. Sample size calculation [64]: for a medium effect size (Cohen’s *w* = 0.30), 80% power, 8 variables included in model, we will need a sample size of *N* = 340. For the LCA, predictors will be selected a priori based on data from aim 1. These predictors will likely include PrEP knowledge, HIV stigma, professional context/environment, and comfort in prescribing. We will use SAS PROC LCA. To find the optimal segmentation, we will fit models with 2–5 classes and compare model fit using Akaike information criterion (AIC), Bayesian information criterion (BIC), and sample-size adjusted BIC. Final model will be selected based on model fit, interpretability, and adequate separation between groups (median posterior probability ≥ 0.70). Interpretability will be based on clinically meaningful groups that lend themselves to implementation strategies to promote the uptake of PrEP among cis women. For utility purposes, it is important to also determine predictors of the latent clusters using variables that are readily available in the public domain. To identify predictors of segment membership, each participant will be assigned to the group with the highest posterior probability of group membership. Predictors of segment membership will include provider/clinic characteristics that are readily available in the public domain: clinician gender, provider type, years in practice, Ryan White funding, region of the country, federally qualified health care center, and community type. We will use multinomial multivariate regression and report OR and 95% CI.

The key output of phase 2 is 2–5 audience segments defined by distinct provider attitudes and knowledge and organizational climates that are predicted by provider and clinic demographics. This rigorously derived information will be used in phase 3 to tailor implementation strategies to each audience segment. Table 2 provides hypothetical audience segments informed by the literature and matched with potential implementation strategies which would go through a refinement process in aim 3.

**Table 2:** Hypothetical audience segments and tailored implementation strategies

*Segment name*	*Example audience segment description*	*Potential targeted implementation strategies*	*Implementation resource intensity*
“Excited and Open”	Consider PrEP in scope of practice; open to prescribing to cis women; low knowledge about cis women specific needs for PrEP; perceived client population at moderate risk; high clinic-level support for PrEP for cis women	Centralizing educational materials to disseminate new data/best practices; provide implementation “how-to” guide for individual provider prescription	Low
“Needs a Nudge”	Does not consider PrEP in current scope of practice; some openness to prescribing; low levels of PrEP knowledge; perceive patients to be at moderate risk of HIV; moderate clinic-level support for PrEP for cis women	Provider level education about HIV risk and PrEP for cis women; provide implementation “how-to” guide for individual provider prescription; provide ongoing consultation; Identify and prepare implementation champions	Moderate
“HIV not a problem for women”	Does not consider PrEP in current scope of practice; not comfortable prescribing; low levels of PrEP knowledge; perceive patients to be at low risk of HIV; high clinic-level support for PrEP for cis women	Provider stigma reduction intervention; Provider level education about HIV among cis women; clinic-level implementation structure developed	High
“Not a Chance”	Does not consider PrEP in current scope of practice; not comfortable prescribing; low levels of PrEP knowledge; perceive patients to be at low risk of HIV; low clinic-level support for PrEP for cis women	Given low levels of provider and clinic-level support for PrEP for women, implementation efforts for this segment are unlikely to yield behavior change in providers. Thus, limited resources should be dedicated to other segments that have a higher likelihood of impact	Too high

### Aim 3 methods

Aim 3 methods consist of iterative expert panels to identify audience segment-specific implementation strategies to facilitate the implementation of PrEP among at-risk cis women. The goal of this phase is to determine the key adoption and implementation outcomes of focus and the associated mechanisms of change (Fernandez’s Task 2) [65]. Then, using the data from aims 1 and 2, and through engagement with expert panels of providers for each audience segment, we will determine and refine a menu of proposed implementation strategies for each audience segment group (Fernandez’s Task 3).

#### Expert panels of providers

We will create a panel of 5–8 cis women’s health providers for each audience segment, comprised of providers representative of that segment from individuals our partner sites. We will maximize geographic and other representational forms diversity within each panel. Each panel of providers will meet at least twice virtually to discuss (1) implementation outcomes and performance objectives, determinants, and change objectives and (2) determination and refinement of implementation strategies.

#### Determination of outcomes, objectives, and determinants

We will utilize an implementation mapping approach in which each audience segment will have its own key PrEP adoption and implementation outcomes. For example, for an audience segment that is characterized by high levels of openness but lack of knowledge about PrEP, appropriate implementation outcomes associated with this audience segment would be focused on provider education about PrEP as a HIV prevention tool and feasibility of prescribing and monitoring PrEP use within the practice (see Table 2). From these implementation outcomes, we will assign performance objectives needed to measure advancement towards those outcomes, such as recommendations for providers to complete trainings on PrEP use among cis women and to gain support from clinic-level stakeholders. From performance objectives, we will ascertain key determinants of those objectives, such as provider PrEP knowledge.

#### Determination and refinement of implementation strategies by audience segment

Following the determination of PrEP implementation outcomes and objectives, we will identify and refine our implementation strategies for each audience segment using an iterative process with each panel of providers utilizing principles of marketing and allowing for feedback from the targeted consumers [66]. The i-PARIHS framework conceptualizes facilitation as a bundle of strategies to improve implementation [67]. Facilitation type(s) will be different for each audience segment depending on the findings of aims 1 and 2. Although actual strategies will be determined through iterative provider engagement, strategies may include provider education, providing on-going consultation, identifying and preparing champions, and creating a learning collaborative [67, 68]. Discussions with providers will include questions on how to prioritize implementation strategies to ensure a balance of impact, achievability, and sustainability.

Output for this phase will consist of the following for each audience segment: (1) proposed implementation outcomes, (2) performance objectives to achieve outcomes, (3) determinants of implementation outcomes (barriers and facilitators, including any behavioral health theories that may accommodate these factors; e.g., Social Cognitive Theory, Theory of Planned Behavior), (4) matrices of change objectives identifying changes in determinants needed to result in achieved performance objectives, and (5) implementation strategies to address change objectives, to be empirically tested in a subsequent project.

## Discussion

The study may encounter a series of logistical challenges. In aim 1, potential challenges relate to the difficulty in scheduling a relatively lengthy interview with medical professionals whose schedules are very limited. For this reason, a wide variety of recruitment methods will be utilized to reach the recruitment goal for this “hard to reach” population. Another aim 1 challenge relates to rapidly coding the qualitative interviews in time to synthetize the findings into a quantitative survey for aim 2. In aim 2, potential challenges will relate to creating a survey that is short enough for hundreds of medical professionals to complete but that will still be comprehensive enough to identify unique audience segments that can have implementation methods tailored to their unique characteristics. Aim 2 challenges will also include recruiting hundreds of busy medical professionals to complete a survey. Strategies such as personalizing e-mail communication, conducting telephone follow-up, and working with professional associations to endorse the survey will be used to help achieve a reasonable response rate. It is possible that the providers surveyed in aim 2 may be too heterogenous (leading to too many individual groups) or too homogenous (leading to only one group).

To address the possibility of heterogeneity, we will limit the LCA to 5 groups. If there is only one group present in aim 2, this is still an important finding, indicating that despite variability in organizational settings and clinical roles, attitudes and other characteristics associated with PrEP prescription are similar. Thus, we will develop of implementation strategies for the single audience segment identified. Demographics may not sufficiently correlate with some latent groups. In this case, implementation efforts in aim 3 will focus on only the groups that can be identified via demographics and additional qualitative work will be undertaken to understand defining characteristics of the latent groups uncorrelated with the demographic information collected prior to implementation strategies identification. Due to selection bias which might affect each of our study aims, the sample might overrepresent providers who are personally committed to PrEP uptake and HIV prevention and consequently produce a suboptimal set of recommendations for the intended population of providers.

## Data Availability

Data sharing is not applicable to this article as this is a protocol paper and no datasets have yet been generated or analyzed.
